# Emerging Relevance of Ghrelin in Programmed Cell Death and Its Application in Diseases

**DOI:** 10.3390/ijms242417254

**Published:** 2023-12-08

**Authors:** Xue Zhang, Zihan Zeng, Yaning Liu, Dan Liu

**Affiliations:** 1 Queen Mary College, Nanchang University, Xuefu Road, Nanchang 330001, China; jp4217120113@qmul.ac.uk (X.Z.); jp4217119205@qmul.ac.uk (Z.Z.); jp4217120117@qmul.ac.uk (Y.L.); 2 School of Pharmacy, Nanchang University, Nanchang 330006, China

**Keywords:** ghrelin, apoptosis, necroptosis, autophagy, pyroptosis, programmed cell death

## Abstract

Ghrelin, comprising 28 amino acids, was initially discovered as a hormone that promotes growth hormones. The original focus was on the effects of ghrelin on controlling hunger and satiation. As the research further develops, the research scope of ghrelin has expanded to a wide range of systems and diseases. Nevertheless, the specific mechanisms remain incompletely understood. In recent years, substantial studies have demonstrated that ghrelin has anti-inflammatory, antioxidant, antiapoptotic, and other effects, which could affect the signaling pathways of various kinds of programmed cell death (PCD) in treating diseases. However, the regulatory mechanisms underlying the function of ghrelin in different kinds of PCD have not been thoroughly illuminated. This review describes the relationship between ghrelin and four kinds of PCD (apoptosis, necroptosis, autophagy, and pyroptosis) and then introduces the clinical applications based on the different features of ghrelin.

## 1. Introduction

Ghrelin is composed of 28 amino acids and was first regarded as an endogenous ligand of the growth hormone secretagogue receptor (GHSR), mainly derived from the fundus of the stomach and pancreas, but exerts effects in various tissues and organs [1,2]. The original role of ghrelin was found to enhance growth hormone secretion [2]. However, with ongoing research, it was gradually discovered to have additional functions, such as producing satiation, regulating metabolism, antagonizing inflammation, modulating depression or anxiety, and so on [3,4]. Two structures of ghrelin, unacylated ghrelin (UAG) and acylated ghrelin (AG), are distributed in the blood and stomach [4]. Abundant UAG exists in the inactive form of ghrelin, which has a poor affinity for GHSR. Ghrelin-o-acyltransferase (GOAT) can convert UAG into the active form AG [5]. Ghrelin is associated with the gastrointestinal tract, cardiovascular system, and other systems [6]. It is proved that in animal models, ghrelin can relieve chronic heart failure, pulmonary hypertension, myocardial infarction, and other pathological conditions, ameliorating cardiac function [7]. According to current studies, ghrelin performs a significant function in glucose and lipid metabolism [3]. However, its concrete role in glucose regulation is still unclear. Ghrelin has been reported to decrease the glucose level [8]. But on account of the obesogenic effects, ghrelin may not be directly applied in diabetes treatment. UAG administration to inhibit the ghrelin/UAG ratio is a potential therapeutic regimen that seems to enhance insulin sensitivity [9]. Meanwhile, blocking the activity of GOAT may be a novel direction for long-term use [9]. As a result, AG might be a preventive or supplementary option for diabetes treatment [10]. The actual clinical application of ghrelin still requires a great deal of efforts. Given the metabolism and affinity of ghrelin, analogs of human ghrelin may be well applied in the treatment. Ghrelin and its analogs have been proven to help in the treatment of multiple diseases such as cancer cachexia, neurodegenerative diseases, and so on. Anamorelin, a GSH-R agonist, has been proven in many studies to exert a ghrelin-like action in the treatment of cancer cachexia [11]. This significant finding dramatically changes the quality of life for cancer patients. In addition, in the rat model of Parkinson’s disease, ghrelin agonist HM01 can alleviate some GI symptoms like constipation [12]. These examples uncovered the application of ghrelin and its agonist in dozens of diseases.

For a long time, PCD and apoptosis have been regarded as equivalent. With the deepening of research, it was found that PCD included apoptosis, pyroptosis, autophagy, necroptosis, ferroptosis, and other forms of cell death [13]. They exert essential effects on the development, evolution, and homeostasis of organisms [14]. Different kinds of PCD have various molecular mechanisms and signaling pathways. The dysregulation of PCD is relevant to multiple diseases, causing aberrant cell death [15]. Several molecules, such as regulators, mediators, and effectors, all participate in these signaling pathways, which have the potential to be therapeutic targets. Consequently, exploring the specific mechanisms of PCD is indispensable for treating multiple diseases, which undoubtedly attracts great attention from researchers worldwide.

Among these PCDs, pyroptosis and necroptosis release inflammatory cytokines, causing inflammation [16,17]. It is found that the anti-inflammation, anti-oxidation, and other features of ghrelin can be essential in inhibiting both pyroptosis and necroptosis. Nevertheless, accumulating evidence demonstrated that ghrelin not only acts on pyroptosis and necroptosis but also regulates autophagy and necroptosis. Ezquerro et al. discovered that ghrelin can promote autophagy, affecting glucose metabolism and maintaining brain function [18]. It has been found that ghrelin can improve pressure-induced tissue injury by inhibiting apoptosis and necroptosis [19]. In nonalcoholic fatty liver disease (NAFLD), ghrelin can influence apoptosis, pyroptosis, and autophagy to improve the deterioration of hepatocytes [20]. Furthermore, the relationship between ghrelin and PCD is more than just inhibition and promotion. The specific role of ghrelin is contingent upon different pathological conditions. The review elaborated on the connection between ghrelin and PCD, elucidating the role of ghrelin in multiple diseases by regulating various forms of PCD including apoptosis, pyroptosis, autophagy, and necroptosis.

## 2. Ghrelin and Apoptosis

Apoptosis is a kind of PCD that is strictly regulated by many factors. It can eliminate the redundant or aberrant cells in multicellular organisms, significantly affecting the normal development and maintenance of homeostasis [21]. Apoptosis is triggered by two unique pathways: intrinsic (mitochondrial) and extrinsic (death receptor) pathways. Intrinsically, it can be triggered by any stimuli that cause oxidative stress, mitochondrial disturbance, and DNA damage, such as ionizing radiation, ischemia, etc. [22]. These stimuli can deliver signals and act on target molecules in the cells. Cytochrome c released from mitochondria into cytosol can combine with apoptotic protease activating factor-1 (Apaf-1) to form a complex called apoptosome, which can activate procaspase-9 to cause apoptosis cascade [23]. The B-cell lymphoma 2 (BCL-2) protein family is involved in regulating the caspase cascade. Intracellular stress can make BH3-only proteins upregulated. These proteins can combine strongly with BCL-2 proteins, thus producing and activating BAX and BAK. They can further boost the release of cytochrome c [23,24]. In the extrinsic pathway, it originated from death receptors, which are part of the tumor necrosis factor (TNF) receptor superfamily including CD27/40, TNFR1/2, and TRAILR [25]. These receptors have cysteine-rich repeats outside and an 80 amino acid protein-binding domain called death domain in the cell, which can bind with specific ligands to recruit adaptor proteins (TRADD and FADD) to the death domain [26,27,28], thus further activating initiator caspases caspase-8 and -10 [29]. Caspase-3 and -7 can be activated as effectors to function well [28]. Both pathways connect through caspase-8-mediated activation of the BH3-only protein BID [30].

A variety of signals can impact the process of apoptosis. As an endogenous brain–gut peptide, ghrelin is expressed primarily in the gut/stomach and minorly in the pancreas [31]. Perhaps it is expressed to a lesser degree in the heart. It participates in various signaling pathways that regulate physiological processes. Apoptosis as a kind of PCD is also regulated by ghrelin. According to previous studies, it is well known that ghrelin has different effects on various types of cells. In other words, ghrelin has the capacity to stimulate or inhibit apoptosis. Occasionally, ghrelin has no action on apoptosis. It is concluded that the selective effect depends on the cells of different organs and the pathological condition of the species [27]. When ghrelin binds to its receptor, ghrelin can mediate the PKA, PKC, and MAP kinase to control the secretion of growth hormone [6]. The JAL/STAT signaling pathway is the leading downstream pathway of growth hormone to perform its function [32].

Ghrelin depresses apoptosis in some types of organ cells, such as cardiomyocytes, neurons, and others. The expression of ghrelin mRNA in cardiomyocytes has been discovered. Ghrelin participates in various aspects of the heart and protects the heart. Endoplasmic reticulum stress (ERS) significantly results in heart diseases. Rat cardiomyocytes exposed to tunicamycin (Tm) or dithiothreitol (DTT) were divided into two ERS models. Ghrelin drastically activated AMPK in cardiomyocytes, and apoptosis of cardiomyocytes was reduced. When employing a selective antagonist of GHS-R1a and an inhibitor of Ca^2+^/Calmodulin-dependent protein kinase kinase (CaMKK), AMPK activation and inhibition of apoptosis were prohibited. Consequently, ghrelin may prevent apoptosis through the GHS-R1a/CaMKK/AMPK pathway [33]. Ghrelin can preserve neonatal rat cardiomyocytes (NRCMs) against hypoxia/reoxygenation (H/R) injury. Under the action of ghrelin, the BCL-2 level can increase while BAX and caspase-3 can decrease.

Furthermore, it can perform effectively by activating the Akt-mTOR signaling pathway [34]. In mouse models of myocardial injury in septic rats, ghrelin has inhibitory effects on apoptosis through the JAK/STAT signaling pathway of growth hormone [35]. Moreover, ghrelin inhibits cardiac cell apoptosis in rats with experimentally induced myocardial infarction by activating Raf-MEK1/2-ERK1/2 signaling [36].

Neuronal apoptosis has been attributed to numerous brain diseases. The blood–brain barrier (BBB) is the protective barrier preventing the entry of some microorganisms, toxins, and macromolecules in order to maintain the homeostasis of the brain [37]. However, the BBB also hinders the delivery of drugs into the brain. It is found that UAG relies on the characteristics of stability to cross the blood–brain barrier (BBB) [38]. The enzyme GOAT existing in the brain can octoacylate UAG into the active form AG to bind GHSRs, which are reported to be distributed in the pituitary and CNS nuclei, such as the arcuate nucleus, hippocampus, and so on [39]. When ghrelin binds to its receptors, it can exert inhibitory effects on neuronal apoptosis [40,41]. Enhancing the ability of ghrelin ligands across the BBB is being explored by researchers [42]. Barros et al. found that ghrelin-loaded liposomes greatly improved the efficiency of delivering ghrelin and guaranteed the activity of released ghrelin [43]. In addition, it has been investigated that polymersomes conjugated with UAG are intended to be delivered to the brain across the BBB [38]. Banks et al. explored the factors influencing the delivery of ghrelin across the BBB in the mouse model. It concluded that serum triglycerides and fasting both facilitate the delivery of ghrelin [44]. Additionally, by analyzing the structures of human and mouse ghrelin, researchers found that the various structures of ghrelin can determine the direction and efficiency of delivery [45]. It is possible that properly altering the structure of ghrelin or its analogs can enhance the delivery across the BBB. These findings all provided new insights into the low delivery efficiency of ghrelin.

In hypoxic-ischemic encephalopathy (HIE), oxidative stress can cause apoptosis. Researchers indicated that the GHSR-1α/AMPK/Sirt1/PGC-1α/UCP2 pathway can contribute to the inhibitory action. The sirt1/PGC-1α pathway is one of the downstream pathways that AMPK activates. The uncoupling protein 2(UCP2) can be regarded as the direct target in the PGC-1α transcriptional regulation. In addition, UCP2 reduces ROS and eliminates ROS in mitochondria [46]. More importantly, UCP2 can mitigate oxidative stress and neuronal apoptosis after damage [47]. In rat models of chronic intraocular hypertension (COH), ghrelin may perform the neuroprotection of retinal neurons by stimulating the ghrelin/GHSR-1a system [48]. It is observed that cleavage products of caspase-3 are inhibited. Ghrelin also performs the same protective function in diabetic encephalopathy [49]. Ghrelin can attenuate high glucose-induced PC12 cell apoptosis and alleviate inflammatory response by modulating the TLR4/NF-κB pathway [49].

In human breast cancer cells, apoptosis can be induced by cisplatin treatment. Apoptosis can be prevented by ghrelin in MDA-MB-231 cells. Ghrelin can alter the levels of BCL-2 and cleaved caspase-3. Additionally, it can initiate the phosphoinositide 3-kinases/Akt/mammalian target of rapamycin (PI3K/Akt/mTOR) signaling pathway [50]. Diabetic cataracts may be induced by high glucose, causing oxidative stress, which can harm human lens epithelial (HLE) cells. Ghrelin also inhibits the loss of cell viability induced by high glucose and HLE cell apoptosis by increasing the expression of BCL-2 and decreasing BAX, which provides novel therapies for ocular diseases [51].

Moreover, ghrelin can preserve the integrity of the cerebral microvascular system during atherosclerosis. The hormone could prevent cerebral microvascular leakage and the decrease of pericytes. In addition to inhibiting the p38 MAPK-JNK pathway, ghrelin can suppress the expression of inflammatory cytokines such as IL-6, MCP-1, and TNF-alpha. This is accomplished by the decreasing level of BAX/Cleaved Caspase-3 and the upregulation of BCL-2 proteins [52]. A far greater body of evidence suggested that ghrelin can protect various types of cells, uncovering the therapeutic effects of ghrelin in different diseases.

In most cases, ghrelin can inhibit apoptosis by influencing distinct pathways. However, some exceptions are found that ghrelin can promote apoptosis. As demonstrated by Sirini et al., they took in vitro maturation (IVM) of bovine oocytes as the research object. Contrary to the previous studies, experimental results suggested that acylated ghrelin can result in cumulus cell death, apoptosis, and DNA damage [53], which differed from the above studies. Furthermore, Ma et al. found that ghrelin could contribute to the apoptosis of MH7A synovial cells and restrain their viability. The cleaved-caspases-8, -9, and -3 levels increased significantly [54]. More intriguingly, it was found that ghrelin cannot affect apoptosis in average rat and human cells, such as human adrenocortical cells, rat osteoblasts, and others [55].

All in all, it can be applied to different aspects of treatment depending on the regulatory function of ghrelin. These findings can provide orientations for therapies for various diseases. Mostly, ghrelin has a beneficial effect on the organisms, which can protect and repair damaged cells. By targeting the direct signaling pathway or specific targets, apoptosis can be inhibited to alleviate diseases. For different cell types, the diverse impacts also have potential significance in the future, which deserves to be explored further (Table 1).

## 3. Ghrelin and Pyroptosis

As a newly identified PCD, pyroptosis can be triggered by pathogen infections [56]. On account of cell swelling and membrane rupture, intracellular contents are released to activate the inflammatory response [24]. Pyroptosis is mediated by caspase-1, which can be activated via various inflammasomes [57]. Additionally, as an essential component of the innate immune system, caspase-1 can generate mature inflammatory cytokines IL-1β and IL-18 from precursors of IL-1β and IL-18. Gasdermin D (GSDMD) makes a difference in the pyroptosis. As a substrate, it can be cleaved with caspase-1 or caspase-11/4/5. Its N-terminal domain can constitute a plasma membrane pore [57,58]. Due to the notable effect, pyroptosis is regarded as gastrin-mediated programmed necrosis [59]. The affected cells can appear in cell swelling, membrane bubbling, and others. The nucleus, however, remains stable.

Based on the activation mechanisms, two signaling pathways result in pyroptosis, which determines whether it depends on caspase-1 [60]. In the canonical pathway, inflammasome assembly is necessary for GSDMD cleavage and IL-1β and IL-18 release. Inflammasomes comprise NOD-like receptors, ASC, and pro-caspase-1 [61]. Inflammasome sensors, also called pattern recognition receptors, can recognize pathogen-associated molecular patterns (PAMP) and danger-associated molecular patterns (DAMP) [62]. There are several common inflammasome sensors: NLRP1, NLRP3, and NLRP4. PYD(pyrin domain) or CARD(caspase activation and recruitment domain) as adaptor proteins can exist in the N terminal, which can recruit pro-caspase-1 [60,63]. Thus, it is activated by caspase-1 [64]. GSDMD can be cleaved into the N terminal and the C terminal. The domain in the N terminal can react with phospholipids on the membrane to form holes. Meanwhile, activated caspase can cleave precursors of IL-1β and IL-18 into mature cytokines to cause inflammatory response [65]. In 2011, Kawasaki et al. provided novel information on caspase-11, which is involved in another non-canonical inflammasome. It is revealed that caspase-11 can mediate the death of mouse macrophages [66]. In the non-canonical pathway, human caspase-4/5 or mouse caspase-11 can recognize lipopolysaccharides to initiate the inflammatory caspases, which can release the cytokines IL-1β and IL-18 [67]. Additionally, the Pannexin-1 channel is widely engaged in interactions between different cells via modulating the transport of ions and ATP [68,69]. The channel is activated by caspase-11 to release ATP, which can act on the P2X7 receptor to mediate the efflux of potassium and activation of NLRP3 inflammasome [70,71]. NLRP3 inflammasome can connect the canonical pathway with the non-canonical signaling pathway.

Several investigations discovered a particular correlation between ghrelin and pyroptosis. However, the underlying mechanisms remain unclear. Currently, the research scope has stayed on the effects of some diseases. Pyroptosis is related to some diseases such as renal, respiratory, and so on [20,72,73]. In the respiratory disease, acute lung injury (ALI) is caused by traumatic brain injury (TBI). In the model of mice with TBI, it is discovered that ghrelin can prohibit pyroptosis, which is mediated by reducing the expression of proinflammatory factors and related proteins. The histology and function of lung tissues can be alleviated by blocking the NF-κB signaling pathway. The significant finding predicts a new treatment direction for TBI-induced ALI [72]. Bronchial asthma is a common respiratory disease. To explore the effect of ghrelin in mice with bronchial asthma, analyzing the expression level of pyroptosis-related proteins in lung tissues is crucial. Experimental results showed that ghrelin can decrease NLRP3, caspase-1, and IL-1β levels. It demonstrates that ghrelin can inhibit pyroptosis to a certain degree, which provides an underlying therapeutic schedule [74].

Substantial findings have shown that ghrelin protects against multiple neurological disorders. Multiple sclerosis is characterized by inflammatory demyelination belonging to autoimmune diseases. In MS patients, GSDMD-mediated pyroptosis may be the reason for inflammatory demyelination [73]. It is demonstrated that ghrelin can prevent pyroptosis by blocking the NLRP3 signaling pathway and decreasing the level of inflammatory cytokines in the model of rats with autoimmune encephalomyelitis [73]. Cheng et al. confirmed that ghrelin can attenuate the activation of NLRP2 inflammasomes and inflammatory response in mice with secondary brain injury (SBI) following intracerebral hemorrhage (ICH) models. In addition, researchers found that ghrelin can reduce oxidative stress and enhance the nuclear factor-E2-related factor 2 (Nrf2)/antioxidative response element (ARE) signaling pathway [75].

The successive activation of inflammasomes like NLRP3 and caspase-1 can mediate pyroptosis. Among these inflammasomes, NLRP3 inflammasomes are the core for inflammatory reactions, suggesting that NLRP3 is highly relevant to pyroptosis [76]. Ghrelin has been discovered to have depressant effects on NLRP3 inflammasomes [77,78,79]. Ling et al. found that ghrelin can inhibit the NLRP3 inflammasome activation and endoplasmic reticulum stress in renal fibrosis caused by unilateral ureteral obstruction [80]. As demonstrated by Chang et al. [78], ghrelin can inhibit the NLRP3 inflammasome by blocking the JAK2-STAT3 and p38 MAPK signaling pathways in the Duchenne muscular dystrophy (DMD) model. Ghrelin can ameliorate inflammatory response to improve muscle function. There is also evidence that ghrelin can reduce oxidative stress and inflammatory reaction in the myocardial ischemia/reperfusion (I/R) injury [77]. In the case of I/R injury, ghrelin can improve myocardial function through the TLR4/NLRP3 signaling pathway [77]. Both studies did not clarify the association between the brain–gut hormone ghrelin and pyroptosis. However, numerous studies regard NLRP3 inflammasome as the essential intermediate in pyroptosis as the underlying effective target.

**Figure 1:**
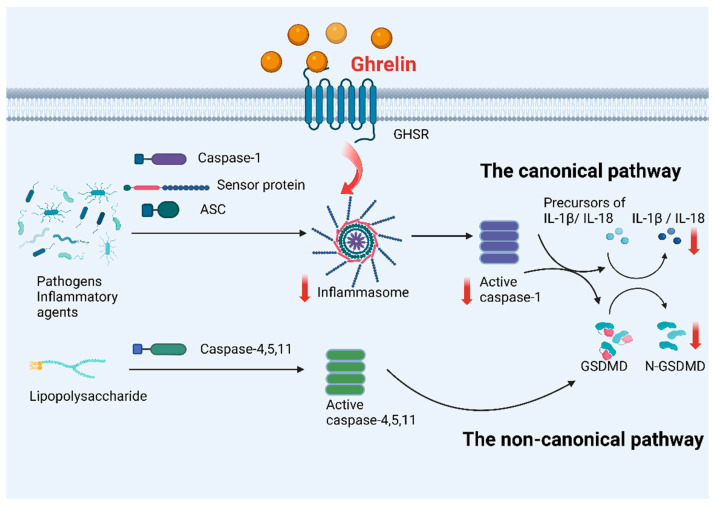
Presentation of the effects of ghrelin on pyroptosis. When ghrelin binds to its receptor, the hormone can inhibit pyroptosis. Pyroptosis is divided into two pathways—the canonical pathway and the non-canonical pathway. In the canonical pathway, ghrelin can decrease the levels of inflammasome consisting of caspase-1, sensor proteins and ACS, active caspase-1, cytokines IL-1β/IL-18, and N-GSDMD. In the non-canonical pathway, the level of N-GSDMD can be affected. These arrows all show that the levels of these molecules decrease. The figure is made using Biorender.

Current research only reveals that ghrelin can inhibit pyroptosis via various signaling pathways in some diseases. Occasionally, the research level is restricted to the anti-inflammatory effect of ghrelin through inhibiting the specific inflammasomes, which did not indicate the direct association between pyroptosis and ghrelin. To summarize, ghrelin can alleviate some diseases, such as TBI, MS, DMD, and others, by inhibiting pyroptosis via various signaling pathways. The hormone can prevent inflammasome activation, which may be related to pyroptosis (Figure 1). The detailed mechanisms are not revealed. However, due to recent research, it has been speculated that ghrelin has anti-inflammatory effects, in which this inflammatory reaction can strongly be related to pyroptosis. In the future, the associations can be well comprehended and applied. Based on the current research, it is inferred that ghrelin may emerge as a novel target in various diseases, opening novel avenues for disease prevention.

## 4. Ghrelin and Autophagy

Autophagy, just as its name implies, is self-eating. This kind of PCD is mediated through the fusion of vesicles harboring cytoplasmic proteins, macromolecules, and organelles with lysosomes to form autolysosomes, which can degrade contents in the vesica to achieve the goal of metabolic requirement and organelle renewal [81,82]. The concept of autophagy was put forward for the first time in 1963 [83]. Yoshinori et al. made enormous contributions to autophagy-related genes (Atgs) and their regulatory mechanisms in 1991 [84]. Numerous scientists have come up with remarkable findings about autophagy, which gave PCD abundant applications in multiple diseases [85,86].

Based on the properties of substances and the method of transferring cargo into lysosomes, autophagy can be divided into three kinds of forms in mammalian cells: microautophagy, macroautophagy, and chaperone-mediated autophagy (CMA) [81]. Microautophagy directly utilizes the invagination of the lysosome membrane, protrusions of the lysosome membrane, or invagination of the late endosome membrane to engulf the cargo, such as organelles, proteins, and so on [87]. There is still inadequate research being conducted on microautophagy. The molecular mechanism and biological function are not fully figured out. Nonetheless, advancements have been made in the area of microautophagy, including micromitophagy, micronucleophagy, and so on [88]. Macroautophagy is the most prevalent autophagy pathway in which autophagosomes containing cytosolic contents fuse with lysosomes [89]. CMA is mediated through the chaperone, which can recognize the proteins carrying specific sequences, convert the folded state into the unfolded state, and then enter the lysosomes through the lysosomal-associated membrane protein 2A (LAMP2A) [90]. Compared with microautophagy and macroautophagy, the degraded proteins are selective.

Because of the complex signaling pathway of autophagy, numerous studies concentrate on the components participating in it. It is reported that mTOR kinase performs the essential function in the process of autophagy [91]. PI3K/Akt signaling pathway and MAPK/Erk1/2 have positive regulations on mTOR, while AMPK and p53 can inhibit the function of mTOR [92]. In addition, ULK1 is also a core component of autophagy, which connects mTOR and AMPK with downstream autophagosomes [93]. It also means that the phosphorylation of ULK1 is controlled with both AMPK and mTOR.

A great deal of evidence shows that ghrelin has an impact on the process of autophagy [93]. Autophagy is closely related to tumors, neurodegenerative diseases, cardiovascular diseases, renal diseases, and so on [59,85]. The associations between ghrelin and autophagy provide novel perspectives into the therapies for these diseases. Some studies have shown that autophagy may exert protective or harmful effects on the diseases. In neurodegenerative diseases, such as Amyotrophic Lateral Sclerosis, Bovine Spongiform Encephalopathy, and so on, accumulating studies show that the inhibition of the autophagy and the mutations of Atgs are associated with these diseases, which result in accumulations of abnormal proteins [94]. Ghrelin may become an underlying treatment through enhancing autophagy, which is dependent on the activation of associated kinases, especially the phospho-AMPK/mTOR axis [18]. Additionally, acylated ghrelin can stimulate the activation of SIRT1 to boost autophagy, which can eliminate harmful proteins [95]. Vascular calcification is always considered to cause cardiovascular diseases [96]. Ghrelin has been demonstrated to ameliorate autophagy by activating AMPK to solve the threat [97]. Intestinal sepsis can cause damage to the small intestinal epithelium, which can be alleviated with ghrelin by boosting the autophagy of damaged epithelial cells [98].

In addition to the stimulative effects on autophagy, the inhibition of autophagy similarly proposes therapies for some diseases that depend on specific pathogenesis. TNF-α secreted from the macrophages can result in the death of hepatocytes, which is involved in nonalcoholic fatty liver disease (NAFLD). It is proved that ghrelin can increase the level of p62 through the AMPK/mTOR pathway, further restraining the hepatic autophagy from treating NAFLD [20]. Chronic obstructive pulmonary disease (COPD) belongs to chronic inflammatory lung disease caused by dysregulated autophagy [99]. It is observed that ghrelin can suppress the inflammation and autophagy. In the meantime, the inhibition of NF-κB and AP-1 signaling is extremely obvious [100]. Ghrelin can bring promising guidance for treating major health problems. In the rat model of chronic intraocular hypertension (COH), ghrelin treatment can decrease the phosphorylation of Akt and mTOR [48], suggesting that ghrelin can inhibit autophagy. Ghrelin can have the effects of neuroprotection in mice with glaucomatous injury. In the rat model of liver fibrosis, the results from the western blotting and qRT-PCR show that ghrelin administration can suppress the TGF-β1/Smad3 as well as NF-κB signaling pathways, indicating autophagy is inhibited to improve liver fibrosis [101].

The application of ghrelin is comprehensive. Not only can it be used to treat various kinds of diseases, but it can also promote the therapeutic effects of some drugs or therapies. Doxorubicin (DOX), commonly used as a tumor chemotherapy agent, has severe cardiotoxicity. DOX can cause cardiomyocyte autophagy and apoptosis [102]. Current research reveals that ghrelin can act as an inhibitor to attenuate autophagy by blocking the effects of AMPK while enhancing the action of p38-MAPK [103]. The significant discovery demonstrated that ghrelin can overcome the drawbacks of DOX. Mesenchymal stem cells (MSCs) can support the cardiac function of patients with ischemia-reperfusion (IR) injury [104]. Sun et al. discovered that ghrelin could improve the efficacy of MSC therapy by enhancing autophagy, accompanied by the upregulation of LC3-II and decrease of P62 proteins [105].

In cancer, autophagy exerts essential effects on the progression of tumors. But in different stages of cancer, autophagy serves various purposes, maybe tumor promotion or tumor suppression [93]. Initially, autophagy as a protection mechanism can inhibit the progression of cancer [106]. However, autophagy can promote tumor cells’ growth and metastasis when entering the intermediate and advanced stages, mediated by epithelial–mesenchymal transition and angiogenesis [107]. It is always considered that inhibition of autophagy is the key to cancer therapies. Nevertheless, other research reveals that inhibition of autophagy does not show satisfactory effects due to weak T-cell responses [108].

On the contrary, enhancing autophagy can strengthen the antitumor T cell responses. Ghrelin can mediate the regulation of autophagy to accomplish the purpose of therapies. It is demonstrated that ghrelin can play a crucial part in activating autophagy, further causing the apoptosis of colorectal adenocarcinoma cells [109]. Although ghrelin provides a new approach in cancer treatment, a deeper comprehension of the molecular mechanisms of tumor cells is indispensable.

**Figure 2:**
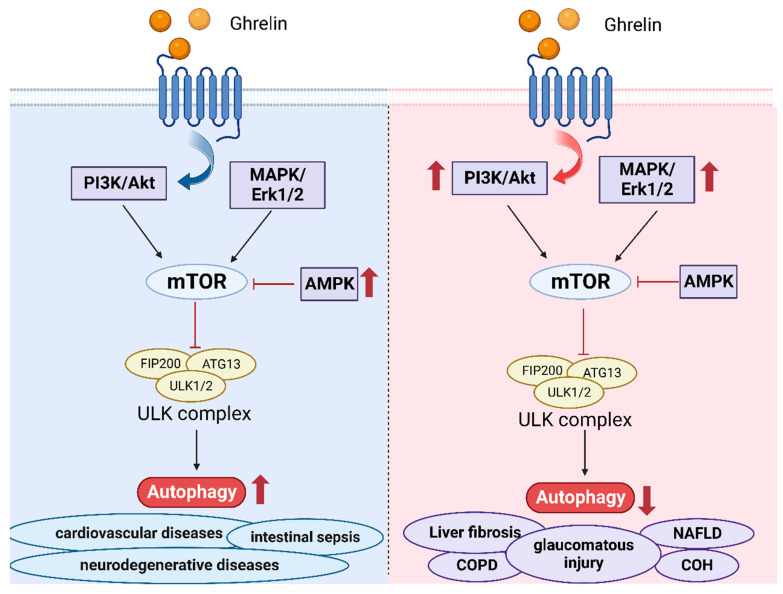
Presentation of the action of ghrelin on autophagy. The effects of ghrelin on autophagy mainly focus on the mTOR/ULK signaling pathway. Ghrelin has positive or negative regulation on autophagy. On the one hand, ghrelin can increase the level of AMPK to enhance autophagy to provide treatment for cardiovascular diseases, intestinal sepsis, and neurodegenerative diseases. On the other hand, ghrelin can prevent autophagy by increasing the levels of PI2K/Akt or MAPK/Erk1/2 signaling pathway, which can be applied in liver fibrosis, COPD, glaucomatous injury, NAFLD, COH, and others. The rising arrow represents the increased levels, while the falling arrow represents the decreased levels. The figure is made using Biorender.

There is a close association between ghrelin and autophagy, which can provide novel directions for cancer, cardiovascular diseases, and so on (Figure 2). Through persistent efforts, it is very possible to fully utilize the more specific effects of ghrelin on human health.

## 5. Ghrelin and Necroptosis

Necroptosis shares characteristics with apoptosis and necrosis, a PCD, and is regarded as a pathway of regulated necrosis [110]. Necroptosis can result in cell death, accompanied by the characteristics of cell swelling, membrane rupture, and breakdown of the cytoplasm and nucleus [111]. Tumor necrosis factor receptor (TNFR) and Toll-like receptor (TLR) family members, intracellular RNA and DNA sensors, and other mediator molecules can stimulate the necroptosis pathway [112]. RIPK1 and RIPK3 are protein kinases that phosphorylate MLKL [113]. The phosphorylation of RIPK3 and MLKL is essential for the key to the necroptosis pathway. It is discovered that RIPK1/3 participates in the transcription of NF-κB, which facilitates the transcription of proinflammatory gene expression [114]. Activated MLKL is capable of the oligomerization and formation of pore complexes to translocate to the plasma membrane, causing the membrane rupture and the release of damage-associated molecular patterns (DAMPs) [114]. When TNF binds to TNFR, the binding can recruit TNFR1-associated death domain protein (TRADD), cellular inhibitors of apoptosis (cIAPs), and linear ubiquitin chain assembly complex (LUBAC), which facilitate the ubiquitination of RIPK1, initiating the mitogen-activated protein kinase (MAPK) pathway [115]. If RIPK1 undergoes the deubiquitination or suppression of cIAPs, caspase-8 can be activated to generate apoptosis [116]. Caspase-8 inhibition is crucial in the formation of necroptosis [116].

What is the relationship between ghrelin and necroptosis? Pressure ulcers are formed due to long-term pressure on the skin or soft tissue, which can increase the mortality of people with pressure ulcers. In one study, researchers used the model of rats with skeletal muscle injury caused by compression to explore the role of ghrelin. Experimental results clearly demonstrate that the levels of RIP1 and RIP3 decreased, which indicated that ghrelin could protect our body through attenuating necroptosis [19]. Current findings may have significant implications for applying the relationship between ghrelin and necroptosis in the treatment of multiple diseases. However, the detailed association between ghrelin and necroptosis still requires further investigation.

However, some findings demonstrated that ghrelin is relevant to the factor TNF-α in the necroptosis pathways. In skin inflammatory diseases, such as psoriasis, contact dermatitis, and others, ghrelin can inhibit the inflammation caused by TNF-α [117]. Ghrelin can attenuate the action of the NF-κB signaling pathway, preventing the release of inflammatory cytokines [117]. The occurrence of osteoarthritis is associated with TNF-α. It was found that ghrelin can improve osteoarthritis by increasing the Akt signaling pathway and inhibiting the NF-κB signaling pathway [118]. In acute myocardial infarction (AMI), ghrelin can attenuate inflammatory reactions with decreased TNF-α [119]. A far greater body of evidence shows the association between ghrelin and TNF-α, which may suggest that ghrelin can influence necroptosis.

Necroptosis involves many diseases, such as neurodegenerative diseases, cancer, inflammatory diseases, and so on [112,120]. With the increasing comprehension of specific molecular mechanisms, the therapies targeted for necroptosis are well applied. Necroptosis may have a multifaced role in cancer, maybe cancer promotion or cancer inhibition [121]. At different stages of cancer progression, the molecular mechanisms are diverse. Kinase inhibitors, metal nanoparticles, necroptosis inducers, and others are standard therapies inhibiting or promoting necroptosis for cancer [120]. In addition to cancer, other cures for diseases fully utilize necroptosis. Unfortunately, the specific molecular mechanisms of necroptosis in various diseases remain poorly understood, and the treatment is still incomplete. Ghrelin is a mysterious hormone with many unimagined functions, such as anti-inflammation and protection [122]. Ghrelin has been proven to have an impact on necroptosis. Therefore, in the future, researchers can pay more attention to the role of ghrelin in necroptosis. Exploring the hormone’s potential and providing a new target for different diseases is feasible.

## 6. Discussion and Conclusions

The review summarized the association between ghrelin and several forms of programmed cell death (PCD), including apoptosis, pyroptosis, autophagy, and necroptosis. PCDs are involved in the formation of organs, tissue remodeling, removal of abnormal cells, and so on [123]. Because of the significance of PCDs, the dysregulation of PCDs can lead to a series of disorders. The link between PCDs and immune system disorders, cancer, cardiovascular disease, and other conditions has been established [124]. Ghrelin can regulate these processes of PCD by controlling the key molecules. In apoptosis, AMPK, Bax, caspase-3, and other factors regulate ghrelin [27,47]. Ghrelin can affect pyroptosis by inhibiting or activating some molecules such as NF-κB, NLRP3, caspase-1, and other proteins [20,73]. During autophagy, the signaling pathways, such as the AMPK/mTOR pathway, NF-κB, AP-1 signaling, and others, can be modulated by ghrelin [48,125]. As for the association between ghrelin and necroptosis, it is not clear, but some studies showed that ghrelin can regulate necroptosis by affecting RIP1 and RIP3 [19]. It is demonstrated that ghrelin can influence the level of TNF-α, which can initiate necroptosis [126]. Furthermore, ghrelin can positively or negatively regulate the various kinds of PCD, depending on the specific diseases. For instance, autophagy has a distinct role in the different stages of cancer.

On the one hand, autophagy can inhibit the progression of cancer. On the other hand, in the late stage of cancer, autophagy can facilitate the severity of cancer [127]. However, ghrelin can always protect our body, clearing harmful cells and maintaining normal cell proliferation. Ghrelin can play crucial roles in various diseases, not just in cancer. Significant evidence shows that ghrelin can become an underlying therapeutic target via inhibiting or promoting the specific signaling pathways of PCDs.

There are a few studies that focus on the association between ghrelin and necroptosis compared to the other three kinds of PCDs. Subsequently, necroptosis deserves greater investigation. In addition, the specific molecular mechanisms of multiple diseases are not entirely worked out. The function of PCD in diseases also attracts our attention. Exploring the association between ghrelin and PCDs can provide promising directions for the broad application of ghrelin in treatment.

**Figure 3:**
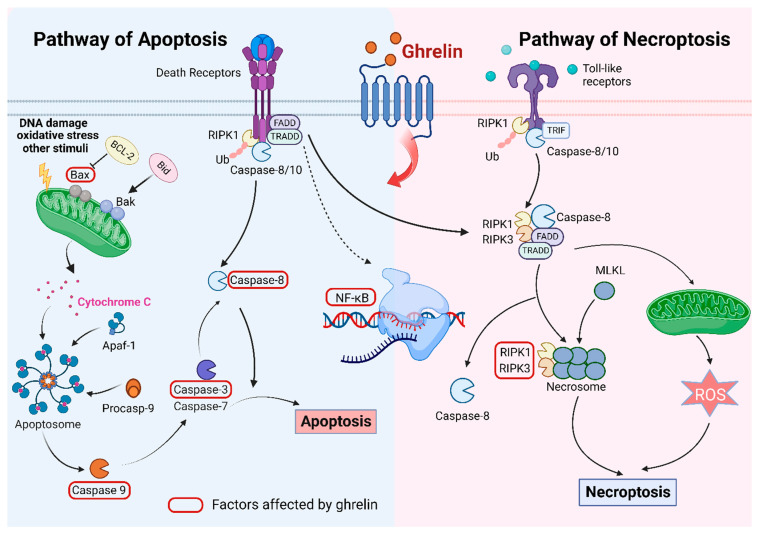
Presentation of association between apoptosis and necroptosis. The pathway of apoptosis is divided into intrinsic (mitochondrial) pathway and extrinsic (death receptor) pathway. In the intrinsic pathway, DNA damage, oxidative stress, and other intrinsic stimuli can stimulate the mitochondria to release cytochrome C, which can combine with Apaf-1 and procaspase-9 to form apoptosomes. Apoptosomes can activate caspase-3/7 to cause apoptosis. The death receptor can initiate apoptosis by caspase-8. In addition, the death receptor can recruit RIPK1/3, FADD, and TRADD to form necrosomes in the absence of caspase-8. ROS can be released from mitochondria to cause necroptosis. Toll-like receptors can also initiate necroptosis. When ligands bind to death receptors, the binding can help the transcription of NF-κB. Death receptors can link apoptosis with necroptosis. When ghrelin binds to its receptor, Bax, caspase 3/8/9 can be affected in the apoptosis. In the necroptosis, ghrelin can influence the levels of RIPK1/3 and NF-κB. BCL-2 can inhibit Bax, while Bid can stimulate Bak. Other arrows represent the following steps. The figure is made using Biorender.

Currently, several findings concentrate only on one specific type of PCD. Nonetheless, since they have a common signaling pathway, many PCDs can communicate with one another. For example, caspase-8 can link apoptosis with necroptosis (Figure 3) [128]. In addition, pyroptosis and necroptosis release inflammatory cytokines such as IL-1β and IL-18, causing inflammatory responses [129]. In most cases, gasdermines (GSDMs) can trigger pyroptosis. However, occasionally, GSDMs can induce apoptosis [130]. Current studies have suggested that the silencing of Atg7 can unexpectedly generate the activation of pyroptosis [131]. Additionally, the relationship between apoptosis and autophagy has been well investigated. They can facilitate each other to enhance cell death. Therefore, since various kinds of PCDs significantly affect the research, we must advance our understanding of the pathogenesis of multiple diseases. Many genes could regulate different types of PCDs. With the continuous deepening of research, an increasing number of PCDs, including ferroptosis and cuproptosis, are being discovered. In subsequent research, further exploration of the emerging PCDs, which can bring unexpected harvests, is warranted. At the same time, the scope can expand into various kinds of PCDs, emphasizing the significance of the common signaling pathways. Connecting ghrelin with specific molecules involved in PCD is a bright direction in treating diseases.

In addition, on account of the characteristics of ghrelin, the majority of recent research has primarily focused on animal models. The clinical application of ghrelin still overcomes a number of difficulties. For example, the hormone has obesogenic properties, which is not recommended for long-time use [132]. In addition, it is well known that AG is the active form that performs biological functions. In the human body, maintaining the activity to exert effects is quite indispensable. Several strategies are proposed to keep the form of AG, such as improving the activity of GOAT, producing the ghrelin analogs [12,41,42]. Nevertheless, these strategies lack maturity. If these problems can be completely solved, ghrelin will open a new avenue in clinical treatment.

All in all, the molecular mechanisms of PCDs in diseases and the regulation of ghrelin on PCDs require further exploration. It is imperative to pay more attention to emerging PCDs and the connections among various PCDs for subsequent research. Ghrelin and ghrelin mimetics can obtain tremendous application prospects in treatment, significantly contributing to human health.

## Figures and Tables

**Table 1:** The summary of effects of ghrelin on various kinds of cells (cardiomyocytes, neurons, human breast cancer cells, human lens epithelial (HLE) cells, pericytes, cumulus cells, MH7A synovial cells, human adrenocortical cells, and rat osteoblasts) and its involved pathway.

Affected Cells	Diseases	Involved Pathways	Effects	Reference
Cardiomyocytes	Heart diseases caused by ERS	GHS-R1a/CaMKK/AMPK pathway	Inhibitory effects	[28]
	Heart diseases caused by H/R injury	Akt-mTOR signaling pathway	inhibitory effects	[29]
	myocardial injury in sepsis	JAK/STAT signaling pathway	Inhibitory effects	[30]
	myocardial infarction	Raf-MEK1/2-ERK1/2 signaling	Inhibitory effects	[31]
Neurons	Hypoxic-ischemic encephalopathy	GHSR-1α/AMPK/Sirt1/PGC-1α/UCP2 pathway	Inhibitory effects	[32]
	COH	Decreasing caspase3	Inhibitory effects	[34]
	diabetic encephalopathy	TLR4/NF-κB pathway	Inhibitory effects	[35]
human breast cancer cells	Breast cancer	PI3K/Akt/mTOR signaling pathway	Inhibitory effects	[36]
human lens epithelial (HLE) cells	Diabetic cataract	Bcl-2 increases Bax decreases	Inhibitory effects	[37]
pericytes	atherosclerosis	p38 MAPK-JNK pathway	Promoting effects	[38]
cumulus cells	-	Unknown	Promoting effects	[39]
MH7A synovial cells	-	cleaved-caspases-8, -9, and -3 increase	Promoting effects	[40]
human adrenocortical cells, rat osteoblasts	-	Unknown	No effects	[41]

## Data Availability

The data are openly available.
